# Notes and News

**Published:** 1890-10-04

**Authors:** 


### NOTES AND NEWS.

COLLECTED AND COMMUNICATED.

THE various medical schools of the metropolis were opened during the current week. At some of the hospitals the usual opening address were delivered, at others a dinner was preferred. This seems a case of "reversion," or progress backwards, unless it be held by the doctors that food for the body is of precedent importance to food for the mind. We shall refer to some of the more noteworthy addresses next week.

HOSPITAL Saturday and Sunday have been very successful, and the amount received altogether will probably show a satisfactory increase over last year's record. The demonstration was confined last year to a Saturday procession of the Friendly and other societies of the district, but this year a church parade on Sunday was added, and will probably continue to form a feature of what is now to be an annual event. The total received from the collections on both days, and from the collecting cards issued by the committee, is expected to exceed £100, which will go to the Surbiton Cottage Hospital and the Kingston Hill Nursing Institute.

THE Toxteth Park Board of Guardians held a special meeting recently to consider the additions to be made to their existing hospital accommodation. It was decided that a smaller scheme than any of those proposed should be proceeded with; that the scheme should be capable of enlargement; that an administration block should be provided; and that the cost should be limited to £16,000. The following resolution was then passed: "That all the plans of the competitors, with copy of the report on each, and the instructions to architects, be sent to the Local Government Board, with a request that they will favour the guardians with an opinion as to their comparative merits, having regard to the regulations of the Local Government Board, and informing the Board that the guardians would desire such a modification of any plan adopted as would limit the cost to a sum of £16,000, or a sum approximating thereto." A motion having been carried that "An expression of opinion be added," the Board mentioned one set of plans as commending themselves to their judgment, surely a not very happy course when all the plans are to be submitted to the judgment of the Local Government Board.

A NEW wing, which has been added to the Wigan Infirmary by Mr. James Henry Johnson, of the Abram Collieries, at a cost of £2,500, was formally opened by the Mayor of Wigan. Afterwards an address of thanks was presented to Mr. Johnson for his handsome gift, the rector, Colonel Blundell, M.P., Mr. Tomlinson, M.P., and other gentlemen taking part in the proceedings. A garden party was held in the grounds by the Mayor and Mayoress, and was attended by a large number of residents.

THE new infectious hospital for Sunderland was open to public inspection last week. It stands in about twelve acres of land, purchased by the Corporation, in Hylton Road. The situation is unexceptionable, being well into the country, and at the same time within easy access of the town. There are five blocks of buildings, namely, what is called the administrative or house department, the laundry block, and three isolated pavilions, into which patients are to be received. The isolated pavilions are divided into two wards each, to contain eight beds; they are heated by hot water pipes, but also by fire stoves in the centre. The hospital was opened on October 1st.

AT a recent quarterly meeting of the Working Men's Hospital Saturday Committee, at Bolton, the chairman announced that the collections this year had been £1,671, or £170 more than the year before. For the past ten years there has been an annual increase of £100, showing that the interests of the Infirmary are being taken to heart by the people at large. Several trades, however, including the cloggers, shoe-makers, and bakers, have made no attempt as a body to aid the movement, and it is somewhat surprising to find that many of the collieries also stand aloof. The weekly payment system appears to be gaining ground, with the result in one instance, of doubling the sum received for the cause.

THE executors of the late Miss Mimpriss (the Trustees, Executors, and Securities Insurance Corporation, Limited) have forwarded cheques for the following legacies bequeathed to the severalinstitutions by Miss Mimpriss' will, viz.: £1,000 to the St. George's Hospital, Hyde Park Corner, £500 each to the Society for Protection of Women and Children, 85, Strand; and the Gordon Hospital, Vauxhall Bridge Road; £200 to the Hospital for Paralysed and Epileptic, Queen's Square, Bloomsbury; £100 each to the Home for Incurable Children, 2, Maida Vale; the Home for Crippled Girls, 17A, Marylebone Road; the All Saints Convalescent Hospital, Eastbourne; the Society for Prevention of Cruelty to Animals; and the Free Cancer Hospital, Fulham Road; and £50 to the Hospital for Consumption, Fulham Road.

NEW premises have been opened for the Hull Orthopædic Hospital, a kind of supplementary specialist institution to the Royal Infirmary. The Orthopædic Hospital was started in 1885, and for the first two years of its existence was undertaken at the private expense of its originator, Dr. Haggard, the honorary surgeon of the institution, but by 1887 it had developed so much that premises had to be taken, whence it was removed the following year to Spencer Street. In consequence of the sale of this latter house, the hospital authorities received notice to quit, and then Miss Liddell, who belongs to a well-known family in Hull, came forward and provided them with the new premises in Wright Street, which she opened the other day at the invitation of the committee. During its brief existence the institution has afforded treatment to some 1,200 cases, and under its new conditions there is no doubt but that it will alleviate much pain and suffering in the large district to which it ministers.

THE following vacancies are announced this week, the amount of the salary in each case being given after the name of the institution :—

**Resident Medical Officers.**

Hospital for Diseases of the Throat, Golden Square, Medical Officer—£50, board, etc.
Cardiff Workhouse, Assistant Medical Officer—£100, board, etc.
Lincoln County Hospital, House Surgeon—£100, board, etc.

**Non-Resident Medical Officers.**

Durham College of Medicine, Demonstrator of Anatomy—£100.
Leeds Public Dispensary, Hon. Ophthalmic Surgeon.
The Hospital for Women, Soho Square, Clinical Assistants.

HOSPITAL SATURDAY at Hastings has again been a most unqualified success. Fine weather contributed greatly to lighten the task of the lady collectors, who took up their station at every likely corner of the streets, and the result has been the excellent sum of £283. Last year the total was £257 7s. 7d., and it is gratifying to note that Hastings shows no exception to the rule that Hospital Saturday collections annually increase in amount.

MRS. LAMBERT, of Sowerby, has built and endowed a cottage hospital at Thirsk, in memory of her husband, son, uncle, and father-in-law, under the name of the Lambert Memorial Hospital. The main part of the building is occupied by two large wards, with nurses' rooms between, each ward having access to a verandah, the surgery, and accident ward. At present preparations are made for five patients only, but this accommodation can readily be increased if necessary. The cost of building has been about £2,000, and the endowment is £7,000, representing an annual income of £220, which it is hoped will render the institution independent of subscriptions. Colonel Dawnay, M.P., performed the opening ceremony, and paid a cordial tribute to Mrs. Lambert's generosity.

BLACKPOOL is at length to have a hospital, and this being so there is no need for us to add to the very plainly worded criticisms of which the town has lately been the object. There can be no doubt that a small institution where accidents can be attended to is an imperative necessity, and as Blackpool is a growing place there is every reason for supposing that the hospital when once begun will grow too. The idea which has been mooted of building a convalescent home in conjunction with the hospital appears to have met with considerable opposition. Yet the most powerful objection to it is that it is premature. Under all the circumstances the Blackpool people may be advised not to be in too much of a hurry. If only care is taken to obtain a sufficiently large site and plans are accepted which admit of extension as necessity arises and funds permit, the hospital may ultimately be large enough and good enough to satisfy its most zealous advocates. For immediate needs a very modest building will suffice. The rest will come in good time.

THE twentieth annual report of the Committee of the Newcastle Hospital Sunday Fund shows some interesting figures. The total collection last year is the largest yet recorded in the town, having been £4,508 12s. 6d. The church and chapel contributions are responsible for £2,089 2s. 2d., and this sum, although larger than that derived from the same source in the two preceding years, is still considerably below what it used to be in the earlier days of the fund's history. The factory and workshop collections, on the other hand, amounted to £2,194 3s. 4d., or 100 guineas more than those in the churches and chapels. Last year the excess on the same side was under £5, although work on the Tyne and in the Newcastle district was more plentiful than it is now. Admission letters, and so forth, received from the various charities and distributed by the committee numbered 7,421, as against 6,280 for the previous year. The report closes with an appeal to the members of congregations to do their utmost to sustain the fund, and to reach once more the excellent totals of former years.

